# Findings of the literature review on larviciding in elimination environments in Asia Pacific

**DOI:** 10.1186/1475-2875-11-S1-P102

**Published:** 2012-10-15

**Authors:** Maxine Whittaker, Moh Seng Chang

**Affiliations:** 1 Asia Pacific Malaria Elimination Network (APMEN), Queensland, Australia; 2 Australian Centre for International and Tropical Health, University of Queensland, Australia

## Background

The Vector Control Working Group of Asia Pacific Malaria Elimination Network posed the question “Do we know enough about the use of larviciding as a vector control method in elimination environments to provide evidence to APMEN Country Partners? This paper summarises our approach to addressing these question and the findings from that literature review.

## Materials and methods

From October 2011 - March 2012, a web based search using the key words: vector control, elimination, malaria, guidelines, standard operating procedures, larviciding, vector management, biological control, was conducted using Google Scholar, PubMed and Scopus. In addition, grey literature was sought through the World Health Organization (WHO) library. A database of literature collected by a research group undertaking a Cochrane Systematic review of vector control was shared with the group. Articles were sought in any language, with abstracts of materials in languages other than English translated by colleagues and members of the vector working group fluent in the required languages, to see if it fulfilled the criteria for inclusion. The date range used for the search was from 1955 - 2012, in order to allow earlier references and manuals regarding larviciding and the use of vector control in the eradication period to be included in the review. In total, 347 articles, books and manuals (12) were reviewed of which 117 met the inclusion criteria.

## Results

There is a large body of literature on a range of larvicides and their suitability for a range of environmental and vectoral contexts that occur in the Asia Pacific region. Very few have been explicitly tested or referred to as suitable in elimination settings nor in many of the Asia Pacific regional countries. Some of these articles, books and guidelines provide useful operational data on the use of larvicides, their safe handling and storage, and other operational details. Only a few discussed monitoring and evaluation aspects of the use of larvicides in programmes. None of the literature reviewed discussed detailed costs, compared cost effectiveness or made cost comparisons between different larvicides and/or between different vector control methods.

## Conclusions

The recent Interim position paper on larviciding in Subsaharan Africa noted that “in general larviciding should be considered for malaria control (with or without other interventions) only in areas where the breeding sites are few, fixed and findable” [1. pg.3]. Although in SSA many of the larval breeding sites were noted not fulfil these three basic criteria for success, in the APMEN region there are some vectoral species that do have these characteristics and are promising vector targets for larval source management. Challenges identified are the lack of published literature operational aspects of larval control/environmental management; although larvicides may not have been considered cost effective in control environments, when moving towards elimination, these remaining larval sources of primary and sometimes the secondary incriminated vectors becomes the “last push”. Without firm evidence it will be hard to convince policy makers and funders to invest in larval source reduction as an elimination strategy.
